# Magnitude of non-communicable disease screening and factors associated with awareness among reproductive age women in Gofa and Basketo zones, Southern Ethiopia: a community-based cross-sectional study

**DOI:** 10.3389/fgwh.2024.1446396

**Published:** 2024-12-19

**Authors:** Markos Manote Domba, Terefe Gelibo Argefa, Abraham Tamirat Gizaw, Abewa Kebede Bitew

**Affiliations:** ^1^ Public Health, Gofa Zone Health Department, South Ethiopia Regional State Health Bureau, Sawla, Ethiopia; ^2^ Public Health Department, Columbia University, Mailman School of Public Health, Addis Ababa, Ethiopia; ^3^ Department of Health, Behavior and Society, Faculty of Public Health, Institute of Health Science, Jimma University, Jimma, Ethiopia; ^4^ Quality Unit, Sawla General Hospital, Sawla, Ethiopia

**Keywords:** non-communicable disease screening, awareness, women, Gofa, Basketo, Ethiopia

## Abstract

**Background:**

The burden of non-communicable diseases (NCDs) increasing at an alarming rate in Ethiopia. NCDs affect reproductive-age women and cause significant threats to future generations. Screening is an important aspect leading to early diagnosis, treatment and preventing the risk of complications and future mortality. However, less attention has been paid in the post-pandemic era of COVID-19. Therefore, this study aims to assess awareness of NCD screening and associated factors among reproductive-age women in the Gofa and Basketo zones.

**Methods:**

A community-based cross-sectional study design was undertaken, employing a multistage cluster sampling method to select participants from the designated zones. Multivariate logistic regression was conducted using Statistical Package for the Social Sciences (SPSS) software. Associations were deemed statistically significant if the *p*-value was ≤0.05.

**Results:**

The awareness level for screening NCDs among women was found to be 54.8%. Specifically, the percentages for awareness of hypertension (HTN), diabetes, cervical cancer, and breast cancer screenings were 52.7%, 42.4%, 38.1%, and 34.8% respectively. However, the study revealed that only 43.0%, 9.4%, 16.2%, and 20.7% of the participants had undergone screening for HTN, diabetes, breast cancer, and cervical cancer respectively. High awareness of NCD screening was significantly associated with urban residence (AOR: 1.68, 95% CI: 1.63, 1.73), Gofa zone residence (AOR: 2.04, 95% CI: 1.95, 2.13), being able to read and write (AOR: 1.06, 95% CI: 1.02, 1.11), having primary (AOR: 1.13, 95% CI: 1.09, 1.16) and secondary school education (AOR: 1.11, 95% CI: 1.00, 1.14), being in the age group of 25–34 (AOR: 1.45, 95% CI: 1.41, 1.49) and ≥35 years (AOR: 1.22, 95% CI: 1.18, 1.26), being married (AOR: 1.25, 95% CI: 1.16, 1.35) and single women (AOR: 1.31, 95% CI: 1.18, 1.43), government employees (AOR: 1.65, 95% CI: 1.54, 1.77), having larger family sizes (>4) (AOR: 1.11, 95% CI: 1.05, 1.12) and having a family member diagnosed with NCD (AOR: 1.16, 95% CI: 1.11, 1.22).

**Conclusions:**

Nearly half of the study population had no awareness of NCD screening and the vast majority had poor screening practice. Strengthening the provision of behavioral change communication strategies through trained health professionals based on the audience's segmentation by age, educational and economic status is needed.

## Introduction

Non-communicable diseases are emerging or rapidly increasing and mainly affect vulnerable groups, which carries a huge burden that impairs the economic and social development of the sub-Saharan Africa (1). Low and middle-income countries undergoing a rapid epidemiological transition characterized by a shift from disease-burden profiles dominated by communicable diseases and childhood illnesses to profiles featuring an increasing pre-dominance of chronic, non-communicable diseases (2). The double burden of non-communicable and communicable diseases poses significant challenges to the populations, health systems, and severely hinders Sustainable Development Goals (3).

NCDs account for almost 65% of women's deaths globally, and the majority of these deaths occur in LMICs (Low and Middle Income Countries) and are premature (4, 5). The reason could be due to an increased exposure to risk factors and limited access to health services in low-income countries (6). In 2016, the prevalence of hypertension (SBP >140 and/or DBP >90 mmHg) among Ethiopian women was 15.6%. About 71% and 96.4% of women had never measured their blood pressure and blood glucose level respectively (7). The large undiagnosed portion of the population with raised blood pressure and blood glucose contributing to the increasing burden of the problem (8).

Ethiopia has guideline for cervical cancer (9) and breast cancer screening (10), and national strategic plan for the prevention and control of major non-communicable diseases (10). The World Health Organization (WHO) and Ethiopian Federal Ministry of Health (FMOH) recommends cervical cancer screening for age eligible women every 3–5 years (11, 12). As per the guidelines, all women aged 20 and above who visit a primary hospital are offered a breast cancer examination (13). Despite existence of national guidelines, screening programs are grossly inadequate or non-available thereby making early detection of precancerous lesions inefficient or in many instances practically impossible, within the countries in the sub-Sahara Africa region including Ethiopia (14).

The findings of a study at urban setting showed that radio and television are the predominant (55.3%) sources of information for cervical cancer screening (15). About 14% of women have regularly engaged in at least one breast cancer screening method (16) and 2.6% of women aged 30–49 had ever undergone screening for cervical cancer (7). A study at northern Ethiopia showed that television, radio and newspaper are major (60%) sources of information for breast cancer screening (17). In underdeveloped nations like Ethiopia, the vast majority of breast cancer patients delay in seeking healthcare (18) and more than three-fourths of cervical cancer patients receive their diagnosis at an advanced stage (19). Patients' delay in seeking care negatively affects treatment effectiveness and survival rate (20). Evidence shows lack of awareness of screening and early diagnosis of diseases significantly affecting utilization of services (18, 21). It is essential to evaluate the awareness of non-communicable diseases (NCDs) screening in the study area. Prior studies in the country have primarily focused on larger cities, ignoring the increasing burden of NCDs in remote and rural areas such as Gofa and Basketo. This study aims to address this gap by assessing the level of screening awareness and its associated factors among reproductive-age women in the Gofa and Basketo zones in Southern Ethiopia.

## Methods

### Study setting and study design

The study was conducted in Gofa and Basketo zones of Southern Ethiopia which are located 464 and 581 KMs respectively to the South of Addis Ababa, capital city of Ehiopia and 143 and 192 KMs respectively from Wolaita Sodo, political and administrative capital of Southern Ethiopia Regional state. The two structures are adjacent and a good representative site for infra-structure limited areas of the region. There are different governmental and private health institutions in the area which includes one General hospital, three primary hospitals, 29 health centers, 214 health posts and 81 private health institutions (i.e., clinics and pharmacies).

Gofa and Basketo zones are administratively divided in to 13 districts (eight rural and five town administrations) having a total population of 720,864 (projected from 2007 Census) in the year 2021. The estimated number of women of reproductive age group is 167,961 (Gofa and Basketo Zone health departments' bi-annual report, 2021). A community based cross-sectional study was employed from Sep 6 to Dec 9/2022.

### Study population

All women aged 15–49 years residing in the Gofa and Basketo zones during the data collection period were eligible for inclusion. This encompassed those who considered the study area their permanent residence for at least 6 months. Exclusions were made for non-permanent residents, pregnant women, individuals institutionalized in hospitals, prisons, nursing homes, or similar facilities, as well as those residing primarily in military camps or dormitories. Additionally, critically ill, mentally disabled, and physically disabled individuals unsuitable for physical participation were excluded.

### Sample size and sampling technique

A mix of sampling approach namely stratified, cluster sampling, systematic random sampling and Kish method were employed to select the study settings and the study participants. Prior to sampling, supervisors and data collectors visited the selected kebeles and conducted a fresh listing of all HHs in that kebele in consultation with local health workers who have a good understanding of the local context. Only one eligible participant in the selected HH was selected using Kish method. Kish method was employed to randomize whom to interview within a household when going door to door and eligible participants in each household were ranked in order of decreasing age (22). The WHO regional office tools for assessing operational district health systems in Africa recommend that for the total number of districts between 10 and 19, sampling 50% of them could be enough (23). Sample size was determined using a single proportion formula considering the *Z*-score = 1.96; Proportion = 50%; marginal error = 0.05; Design effect = 3.35; and non-response rate = 10%, making the total sample size of 1,416 respondents.

### Study variables and measurement

#### Dependent variables

Non-communicable disease screening include: screening for diabetes mellitus, hypertension, cervical cancer or breast cancer. Hypertension screening, diabetes screening, cervical cancer and breast cancer screening were measured with questions: Have you ever had your blood pressure measured by a doctor or other health worker? Have you ever had your blood sugar measured by a doctor or other health worker? Have you ever checked for cervical cancer? Have you ever examined for breast cancer? Variables for evaluating awareness of hypertension screening, diabetes screening, cervical cancer and breast cancer screening were measured with questions: Have you ever heard or read about hypertension screening? Have you ever heard or read about diabetes screening? Have you ever heard or read about cervical cancer screening? Have you ever heard or read about breast cancer examination? The measurements were based on women's self-report and the awareness of screening services were measured dichotomously; yes (if a woman heard or read about screening services) vs. no (if a woman has not heard or read about screening services).

#### Independent variable

Socio-demographic and cultural variables include: age, place of residence, family history, family size, educational status, marital status, occupation of women, social influence to NCD screening (the role of participants' social network members in encouraging them to get screening services) and wealth status of household. Wealth Status was derived from the wealth index (five quintiles in the data set; poorest, poor, middle, rich and the richest) for the households. The variables included to calculate the index were main material of the walls, roofing, floor, separate room for cooking, type of fuel household mainly use for cooking, kind of toilet facility household use, household's ownership of phone, radio, Television, mattress, bed, watch, stove, table, chair, beehive, ox, caw, hen, motorcycle and Generator (24, 25). Knowledge related factors/variables include getting advice from health professionals and using mass media. Structural factors include membership in a functional women's development army.

### Data collection tool and method

The data collection instrument is a questionnaire developed with adaptation of the WHO Stepwise Surveillance questionnaire (26). This questionnaire was translated into Gofatho and Basket languages and subsequently back-translated into English to ensure accuracy. Socio demographic, knowledge and health system related data were collected. Data collectors were Health Extension Workers who had two days training on collecting data from all the three Steps before the survey. The survey data was collected using the hard copy and the average time for each interview was 20 min.

### Data quality management

To maintain data quality, the questionnaire underwent translation and pretesting as necessary. Following data collection, thorough checks for completeness and consistency were conducted, and coding was performed by both the supervisor and principal investigator.

### Weighting of data

Data was weighted because it comprises sample of target population. The sample weighting was carried out to correct differences in distribution of the sample age and area of residence vs. the target population and probabilities of selection. The sample weight for each case in the sample accounts for the number of cases in the sampling frame. The product of the sample weight was used in weighted analysis.

### Data analysis

Using data exported to the SPSS software version, the descriptive statistics were done and summarized by frequencies and proportion for categorical predictors. A series of bivariate comparisons were made prior to any multiple regression modeling. Binary logistic regression was used to see the association between the outcome and independent variables and variables with a *p*-value of less than 0.25 in the binary logistic regression were fitted into a multivariable logistic regression model to control for confounding effects. The strength of the association was estimated by odds ratio and its 95% confidence interval. Associations with a *p*-value ≤ 0.05 are considered statistically significant.

### Ethical considerations

The survey protocol obtained ethical clearance from the institution review boards of the Southern Regional State Public Health Institute and Selinus University of Science and Literature. A paper-based written informed consent form was administered to eligible participants. At each stage of the process, consent was indicated by signing or making a mark on the consent form on a printed copy, which was retained by the participant and the data collector. A designated head of household provided written consent for the household to take part in the survey, after which individual members were rostered during a household interview. For minors ages 15–17, parents/guardians provided permission which was followed by assent by the participant.

## Results

### Socio-demographic characteristics of participants

The average age of participants was 28.9 years with a standard deviation of 7.5 years, and 41.5% fell within the 25–34 age range. A significant portion (39%) had no formal education, while the majorities (93.4%) were married. Predominantly, participants hailed from the Gofa zone (93.3%) and rural areas (83.3%). Furthermore, 84% resided in rural regions. In terms of wealth distribution, approximately 20.5%, 21.1%, and 20.6% of participants were categorized into the poorest, poorer, and middle wealth quintiles respectively. Housewives comprised the largest occupational group, accounting for 72% of participants. Additional socio-demographic details can be found in Table 1.

**Table 1:** Socio-demographic characteristics of participants.

Characteristics	Un-weighted count	Weighted percent
Age		
15–24	400	29.4
25–34	599	41.5
≥35	405	29.1
Educational status		
Illiterate	519	38.6
Able to read and write	147	9.6
Primary education	400	28.7
Secondary education and above	338	23.1
Marital status		
Married	1,297	93.4
Single	68	4.5
Widowed/divorced	39	2.1
Residence		
Urban	393	16.2
Rural	1,011	83.8
Zone		
Gofa	1,244	93.3
Basketo	160	6.7
Wealth index		
Poorest	288	20.5
Poorer	286	21.1
Middle	277	20.6
Richer	280	19.5
Richest	273	18.3
Occupational status		
Housewife	981	71.9
Merchant	172	12.7
Government employee	76	4.4
Other	175	11.0
Family size		
≤4	544	38.4
>4	860	61.6
Family history of NCDs		
No	1,292	92.7
Yes	112	7.3
Social influence to NCDs screening		
No	1,064	75.5
Yes	340	24.5

Others in occupational status: daily laborers, students and maidservants.

### Non-communicable disease screening and awareness

**Figure 1:**
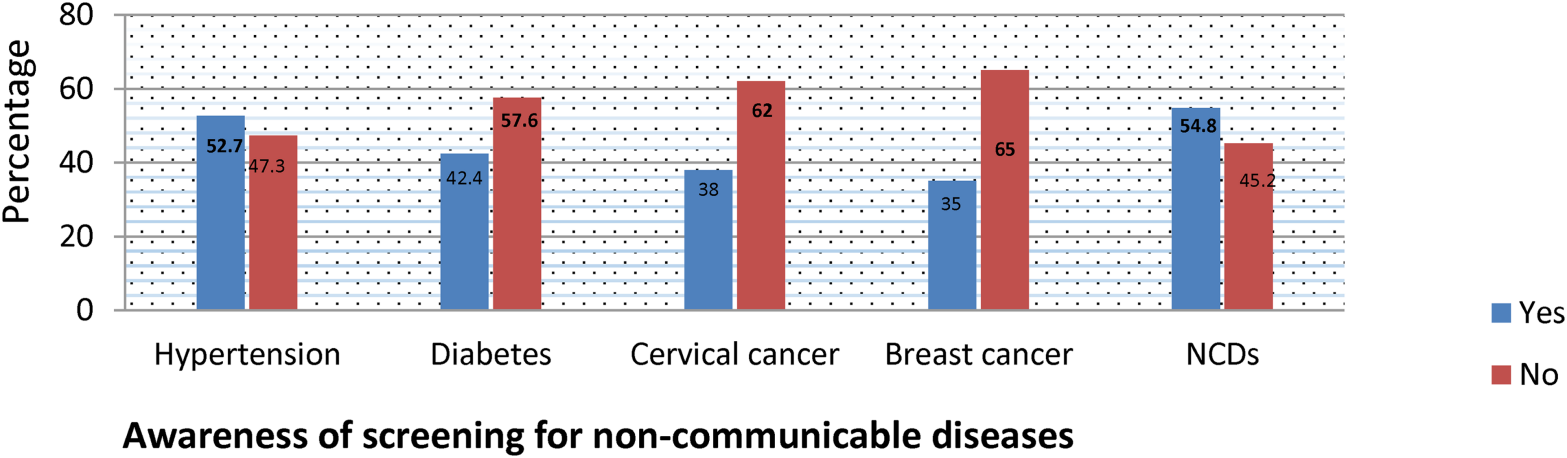
Awareness of non-communicable diseases screening among reproductive age women in Gofa and Basketo zone, Southern Ethiopia.

About 52.7% of the study participants were aware of screening for Hypertension; and 43.0% of them screened for hypertension. Overall prevalence of awareness of screening for diabetes was 42.4%; but only 9.4% of the study participants were screened for diabetes. Thirty eight percent of the study participants were aware of cervical cancer screening. However, the service utilization among women was only 16.2%. Despite 35% of the study participants were aware of breast cancer screening, only 20.7% screened for breast cancer (including BSE and/or clinical examination). About 45.2% of the study participants were not aware of NCD screening (Figure 1).

### Factors associated with awareness of NCD screening

The detailed factors associated with awareness of hypertension, diabetes, cervical cancer, breast cancer and NCD screening are presented in Tables 2–6.

**Table 2:** Factors associated with awareness of hypertension screening among reproductive age women.

Variables	Aware of HTN screening (%)	COR (95% CI)	*P*-value	AOR 95% CI	*P*-value
Zone					
Gofa	690 (53.8%)	**1.94** (**1.86, 2.02)**	**<0**.**001**	**2.08** (**1.99, 2.17)**	**<0**.**001**
Basketo	60 (37.5)	1		1	
Residence					
Urban	267 (66.8)	**2** (**1.96, 2.10)**	**0**.**001**	**1.74** (**1.69, 1.79)**	**<0**.**001**
Rural	483 (49.9)	1		1	
Age					
15–24	201 (48.6)	1		1	
25–34	334 (55.9)	**1.34** (**1.31, 1.37)**	**<0**.**001**	**1.40** (**1.36, 1.44)**	**<0**.**001**
>35	215 (52.1)	**1.15** (**1.12, 1.18)**	**<0**.**001**	**1.19** (**1.15, 1.23)**	**<0**.**001**
Educational level					
Illiterate	261 (51.5)	1		1	
Read and write	80 (52.2)	1.03 (0.99, 1.07)	.140	0.98 (0.95, 1.02)	0.442
Primary education	212 (52.4)	**1.04** (**1.01, 1.06)**	**0**.**007**	**1.12** (**1.08, 1.15)**	**<0**.**001**
Secondary education	197 (55.1)	**1.16** (**1.13, 1.19)**	**<0**.**001**	1.02 (0.99, 1.06)	0.151
Marital status					
Married	686 (52.2)	**0.87** (**0.81, 0.94)**	**0**.**001**	**1.13** (**1.05, 1.22)**	**0**.**002**
Single	41 (60.1)	**1.2** (**1.1, 1.3)**	**0**.**001**	**1.28** (**1.16, 1.40)**	**<0**.**001**
Widowed divorced	23 (55.6)	1		1	
Occupation					
Housewife	499 (50.9)	**0.65** (**0.63, 0.67)**	**0**.**001**	**0.64** (**0.62, 0.67)**	**<0**.**001**
Merchant	82 (48.0)	**0.58** (**0.55, 0.6)**	**0**.**001**	**0.58** (**0.55, 0.61)**	**<0**.**001**
Gov't Employee	60 (72.7)	**1.7** (**1.6, 1.8)**	**0**.**001**	**1.99** (**1.86, 2.13)**	**<0**.**001**
Other	109 (61.6)	1		1	
Family size					
<4 dwellers	289 (51.3)	**0.92** (**0.89, 0.94)**	**0**.**001**	**0.90** (**0.88, 0.93)**	**<0**.**001**
>4dwellers	461 (53.5)	1		1	
Wealth status					
Poorest	151 (51.5)	**0.86** (**0.84, 0.89)**	**0**.**001**	1.00 (0.96, 1.03)	0.979
Poor	144 (50.7)	**0.84** (**0.81, 0.87)**	**0**.**001**	**0.96** (**0.93, 0.99)**	**0**.**041**
Middle	155 (55.7)	1.02 (0.99, 1.06)	0.149	**1.09** (**1.05, 1.13)**	**<0**.**001**
Rich	146 (50.6)	**0.83** (**0.81, 0.86)**	**0**.**001**	**0.86** (**0.83, 0.89)**	**<0**.**001**
Richest	154 (55.1)	1		1	

COR, crude odds ratio: odds ratio by bivariate analysis. 95% CI, confidence interval at the 95% level.

AOR, adjusted OR, odds ratio by multiple logistic regression 1: Referent category.

Bold values indicate statistical significance at *p*-value ≤0.05.

**Table 3:** Factors associated with awareness of diabetes screening among reproductive age women.

Variables	Aware of diabetes screening (%)	COR (95% CI)	*P*-value	AOR (95% CI)	*P*-value
Zone					
Gofa	577 (44.0)	**0.32** (**0.30, 0.33)**	**<0**.**001**	**3.26** (**3.09, 3.44)**	**<0**.**001**
Basketo	32 (20.0)	1		1	
Residence					
Urban	248 (61.8)	**2.6** (**2.5, 2.6)**	**0**.**001**	**2.11** (**2.05, 2.18)**	**<0**.**001**
Rural	361 (38.6)	1		1	
Age					
15–24	163 (38.9)	1		1	
25–34	265 (44.4)	**1.25** (**1.22, 1.29)**	**<0**.**001**	**1.31** (**1.27, 1.34)**	**<0**.**001**
≥35	181 (43.1)	**1.18** (**1.15, 1.22)**	**<0**.**001**	**1.23** (**1.19, 1.28)**	**<0**.**001**
Education level					
Illiterate	208 (40.3)	1		1	
Read and write	67 (42.3)	**1.09** (**1.05, 1.13)**	**<0**.**001**	0.99 (0.96, 1.03)	0.793
Primary education	169 (41.8)	**1.06** (**1.04, 1.09)**	**<0**.**001**	**1.18** (**1.14, 1.21)**	**<0**.**001**
Secondary education	165 (46.7)	**1.31** (**1.26, 1.33)**	**<0**.**001**	**1.20** (**1.16, 1.24)**	**<0**.**001**
Marital status					
Married	562 (42.3)	**1.18** (**1.1, 1.27)**	**0**.**001**	**1.54** (**1.42, 1.67)**	**<0**.**001**
Single	29 (46.7)	**1.40** (**1.30, 1.50)**	**0**.**001**	**1.53** (**1.39, 1.69)**	**<0**.**001**
Widowed/divorced	18 (38.3)	1		1	
Occupation					
Housewife	408 (41.2)	**0.67** (**0.65, 0.69)**	**0**.**001**	**0.66** (**0.64, 0.69)**	**<0**.**001**
Merchant	61 (35.3)	**0.52** (**0.5, 0.54)**	**0**.**001**	**0.53** (**0.51, 0.56)**	**<0**.**001**
Gov't employee	49 (60.7)	**1.46** (**1.38, 1.56)**	**0**.**001**	**1.70** (**1.59, 1.82)**	**<0**.**001**
Other	91 (51.2)	1		1	
Family size					
≤4 dwellers	230 (40.7)	**0.89** (**0.87, 0.91)**	**0**.**001**	**0.84** (**0.82, 0.86)**	**<0**.**001**
>4dwellers	379 (43.5)	1		1	
Wealth status					
Poorest	125 (41.2)	**0.76** (**0.74, 0.79)**	**0**.**001**	**0.90** (**0.87, 0.94)**	**<0**.**001**
Poor	115 (40.6)	**0.75** (**0.73, 0.78)**	**0**.**001**	**0.87** (**0.84, 0.90)**	**<0**.**001**
Middle	118 (42.6)	**0.81** (**0.78, 0.83)**	**0**.**001**	**0.87** (**0.84, 0.91)**	**<0**.**001**
Rich	116 (40.0)	**0.73** (**0.70, 0.75)**	**0**.**001**	**0.76** (**0.74, 0.79)**	**<0**.**001**
Richest	135 (47.9)	1		1	
Do you use mass media?					
No	373 (40.5)	1		1	
Yes	236 (46.5)	**1.28** (**1.25, 1.31)**	**<0**.**001**	**1.07**(**1.04, 1.10)**	**<0**.**001**

COR, crude odds ratio: odds ratio by bivariate analysis, 95% CI, confidence interval at the 95% level.

AOR, adjusted OR: odds ratio by multiple logistic regression 1: referent category.

Bold values indicate statistical significance at *p*-value ≤0.05.

**Table 4:** Factors associated with awareness of cervical cancer screening among reproductive age women.

Variables	Aware of cervical cancer screening (%)	COR (95% CI)	*P*-value	AOR 95% CI	*P*-value
Zone					
Gofa	530 (40.1)	**6.03** (**5.63, 6.45)**	**<0**.**001**	**6.15** (**5.74, 6.59)**	**<0**.**001**
Basketo	16 (10.1)	1		1	
Residence					
Urban	226 (57.6)	**2.6** (**2.5, 2.7)**	**0**.**001**	**2.11** (**2.03, 2.16)**	**<0**.**001**
Rural	320 (34.3)	1		1	
Age					
15–24	140 (33.7)	1		1	
25–34	246 (41.4)	**1.39** (**1.36, 1.43)**	**<0**.**001**	**1.51** (**1.45, 1.54)**	**<0**.**001**
≥35	160 (37.8)	**1.19** (**1.16, 1.23)**	**0**.**001**	**1.32** (**1.27, 1.36)**	**<0**.**001**
Education level					
Illiterate	180 (34.0)	1		1	
Read and write	65 (40.7)	**1.33** (**1.28, 1.39)**	**<0**.**001**	**1.26** (**1.21, 1.31)**	**<0**.**001**
Primary education	159 (39.8)	**1.28** (**1.25, 1.32)**	**<0**.**001**	**1.48** (**1.44, 1.53)**	**<0**.**001**
Secondary education	142 (41.7)	**1.39** (**1.35, 1.43)**	**<0**.**001**	**1.46** (**1.41, 1.52)**	**<0**.**001**
Marital status					
Married	503 (37.8)	0.96 (0.88, 1.03)	0.23	**1.15** (**1.06, 1.24)**	**0**.**001**
Single	27 (44.8)	**1.30** (**1.20, 1.40)**	**0**.**001**	**1.64** (**1.48, 1.81)**	**<0**.**001**
Widowed/divorced	16 (38.8)	1		1	
Occupation					
Housewife	374 (37.4)	**0.74** (**0.72, 0.77)**	**0**.**001**	**0.82** (**0.79, 0.86)**	**<0**.**001**
Merchant	57 (32.4)	**0.6** (**0.57, 0.62)**	**0**.**001**	**0.66** (**0.63, 0.69)**	**<0**.**001**
Gov't Employee	36 (50.2)	**1.3** (**1.18, 1.33)**	**0**.**001**	**1.58** (**1.48, 1.69)**	**<0**.**001**
Other	79 (44.6)	1		1	
Family size					
≤4 dwellers	205 (36.4)	**0.9** (**0.87, 0.91)**	**0**.**001**	**0.82** (**0.81, 0.84)**	**<0**.**001**
>4dwellers	341 (39.2)	1		1	
Wealth status					
Poorest	110 (36.4)	**0.72** (**0.69, 0.75)**	**0**.**001**	**0.84** (**0.81, 0.88)**	**<0**.**001**
Poor	107 (36.9)	**0.74** (**0.72, 0.77)**	**0**.**001**	**0.83** (**0.81, 0.86)**	**<0**.**001**
Middle	106 (39.7)	**0.83** (**0.8, 0.86)**	**0**.**001**	**0.90** (**0.87, 0.93)**	**<0**.**001**
Rich	96 (33.7)	**0.64** (**0.62, 0.66)**	**0**.**001**	**0.67** (**0.65, 0.69)**	**<0**.**001**
Richest	127 (44.2)	1		1	

COR, crude odds ratio: odds ratio by bivariate analysis, 95% CI, confidence interval at the 95% level.

AOR, adjusted OR: odds ratio by multiple logistic regression 1: referent category.

Bold values indicate statistical significance at *p*-value ≤0.05.

**Table 5:** Factors associated with awareness of breast cancer screening among reproductive age women.

Variables	Aware of breast cancer screening (%)	COR(95% CI)	*P*-value	AOR 95% CI	*P*-value
Zone					
Gofa	492 (36.6)	**5.20** (**4.86, 5.57)**	**<0**.**001**	**5.64** (**5.26, 6.05)**	**<0**.**001**
Basketo	16 (10.0)	1		1	
Residence					
Urban	219 (55.1)	**2.7** (**2.6, 2.8)**	**0**.**001**	**2.29** (**2.23, 2.36)**	**<0**.**001**
Rural	289 (30.9)	1		1	
Age					
15–24	132 (30.8)	1		1	
25–34	227 (38.0)	**1.38** (**1.34, 1.42)**	**<0**.**001**	**1.47** (**1.43, 1.52)**	**<0**.**001**
≥35	149 (34.4)	**1.18** (**1.14, 1.21)**	**<0**.**001**	**1.26** (**1.21, 1.30)**	**<0**.**001**
Education level					
Illiterate	168 (31.6)	1		1	
Read and write	58 (35.5)	**1.19** (**1.15, 1.24)**	**<0**.**001**	**1.08** (**1.04, 1.13)**	**<0**.**001**
Primary education	149 (36.4)	**1.24** (**1.20, 1.27)**	**<0**.**001**	**1.32** (**1.28, 1.36)**	**<0**.**001**
Secondary education	133 (38.1)	**1.33** (**1.29, 1.37)**	**<0**.**001**	**1.22** (**1.17, 1.26)**	**<0**.**001**
Marital status					
Married	466 (34.4)	1.1 (0.99, 1.16)	0.08	**1.40** (**1.29, 1.52)**	**<0**.**001**
Single	27 (44.8)	**1.70** (**1.50, 1.80)**	**0**.**001**	**2.17** (**1.96, 2.40)**	**<0**.**001**
Widowed/Divorced	15 (32.9)	1		1	
Occupation					
Housewife	344 (33.9)	**0.7** (**0.65, 0.69)**	**0**.**001**	**0.75** (**0.72, 0.78)**	**<0**.**001**
Merchant	48 (27.6)	**0.5** (**0.48, 0.52)**	**0**.**001**	**0.56** (**0.53, 0.59)**	**<0**.**001**
Gov't Employee	37 (49.3)	**1.3** (**1.2, 1.35)**	**0**.**001**	**1.75** (**1.64, 1.87)**	**<0**.**001**
Other	79 (43.4)	1		1	
Family size					
≤4 dwellers	193 (33.3)	**0.9** (**0.87, 0.91)**	**0**.**001**	**0.83** (**0.81, 0.85)**	**<0**.**001**
>4dwellers	315 (35.8)	1		1	
Wealth status					
Poorest	106 (34.4)	**0.87** (**0.84, 0.89)**	**0**.**001**	1.02 (0.98, 1.06)	0.319
Poor	98 (33.9)	**0.85** (**0.82, 0.88)**	**0**.**001**	0.98 (0.95, 1.02)	.348
Middle	100 (36.3)	**0.94** (**0.91, 0.98)**	**0**.**001**	1.04 (0.99, 1.07)	.058
Rich	93 (32.2)	**0.78** (**0.76, 0.81)**	**0**.**001**	**0.82** (**0.79, 0.86)**	**<0**.**001**
Richest	111 (37.7)	1		1	
Time taken from home to the health facility					
<1 h	311 (35.2)	**0.94** (**0.90, 0.98)**	**0**.**009**	**1.23** (**1.17, 1.29)**	**<0**.**001**
1–2 h	158 (33.9)	**0.89** (**0.85, 0.94)**	**0**.**001**	**1.11** (**1.06, 1.17)**	**<0**.**001**
>2 h	39 (36.5)	1		1	

COR, crude odds ratio: odds ratio by bivariate analysis, 95% CI, confidence interval at the 95% level.

AOR, adjusted OR: odds ratio by multiple logistic regression 1: referent category.

Bold values indicate statistical significance at *p*-value ≤0.05.

**Table 6:** Factors associated with awareness of non-communicable disease screening among reproductive age women.

Variables	Aware of NCD screening (%)	COR (95% CI)	*P*-value	AOR 95% CI	*P*-value
Zone					
Gofa	715 (55.9)	**1.91** (**1.82, 1.98)**	**<0**.**001**	**2.02** (**1.93, 2.11)**	**<0**.**001**
Basketo	64 (40.0)	1		1	
Residence					
Urban	272 (68.2)	**1.97** (**1.9, 2.03)**	**0**.**001**	**1.68** (**1.63, 1.73)**	**<0**.**001**
Rural	507 (52.2)	1		1	
Age					
15–24	210 (50.6)	1		1	
25–34	346 (58.3)	**1.37** (**1.33, 1.40)**	**<0**.**001**	**1.45** (**1.41, 1.49)**	**<0**.**001**
≥35	223 (54.0)	**1.14** (**1.11, 1.18)**	**<0**.**001**	**1.23** (**1.19, 1.27)**	**<0**.**001**
Education level					
Illiterate	269 (53.2)	1		1	
Read and write	85 (55.5)	**1.11** (**1.06, 1.14)**	**0**.**001**	**1.06** (**1.02, 1.11)**	**0**.**002**
Primary education	218 (54.1)	**1.04** (**1.01, 1.07)**	**0**.**003**	**1.15** (**1.11, 1.18)**	**<0**.**001**
Secondary education	207 (58.2)	**1.22** (**1.19, 1.26)**	**0**.**001**	**1.13** (**1.09, 1.17)**	**<0**.**001**
Marital status					
Married	714 (54.5)	0.95 (0.88, 1.03)	0.196	**1.25** (**1.16, 1.35)**	**<0**.**001**
Single	42 (61.1)	**1.25** (**1.20, 1.40)**	**0**.**001**	**1.31** (**1.19, 1.44)**	**<0**.**001**
Widowed/divorced	23 (55.7)	1		1	
Occupation					
Housewife	516 (52.7)	**0.62** (**0.60, 0.64)**	**0**.**001**	**0.61** (**0.59, 0.64)**	**<0**.**001**
Merchant	90 (52.4)	**0.61** (**0.60, 0.64)**	**0**.**001**	**0.61** (**0.58, 0.64)**	**<0**.**001**
Gov't employee	60 (72.7)	**1.50** (**1.40, 1.60)**	**0**.**001**	**1.63** (**1.52, 1.75)**	**<0**.**001**
Other	113 (64.2)	1		1	
Family size					
≤4 dwellers	301 (53.6)	**0.92** (**0.9, 0.94)**	**0**.**001**	**0.92** (**0.91, 0.95)**	**<0**.**001**
>4dwellers	478 (55.6)	1		1	
Family member living with NCD					
No	708 (54.2)	**0.72 (0.70, 0.75)**	**0**.**001**	**0.85** (**0.81, 0.89)**	**<0**.**001**
Yes	71 (62.3)	1		1	
Wealth status					
Poorest	156 (53.5)	**0.81** (**0.79, 0.84)**	**0**.**001**	**0.95** (**0.92, 0.99)**	**0**.**007**
Poor	150 (52.6)	**0.78** (**0.76, 0.81)**	**0**.**001**	**0.91** (**0.88, 0.94)**	**<0**.**001**
Middle	159 (57.0)	**0.94** (**0.91, 0.97)**	**0**.**001**	1.01 (0.97, 1.04)	.706
Rich	151 (52.8)	**0.79** (**0.77, 0.82)**	**0**.**001**	**0.83** (**0.81, 0.86)**	**<0**.**001**
Richest	163 (58.5)	1		1	
Time taken from home to the health facility					
<1 h	474 (54.2)	**0.87** (**0.83, 0.91)**	**0**.**001**	0.99 (0.95, 1.034)	0.714
1–2 h	247 (55.4)	**0.91** (**0.87, 0.95)**	**0**.**001**	1.05 (0.99, 1.11)	0.056
>2 h	58 (57.7)	1		1	

COR, crude odds ratio: odds ratio by bivariate analysis, 95% CI, confidence interval at the 95% level.

AOR, adjusted OR: odds ratio by multiple logistic regression 1: referent category.

Bold values indicate statistical significance at *p*-value ≤0.05.

### Factors associated with awareness of hypertension screening

In the present study, 52.7% of the study participants were aware of screening for Hypertension. Women in Gofa Zone were more aware of screening for HT (53.8%) than women living in Basketo (37.5%). This difference is statistically significant. Women in Gofa Zone (AOR: 2.08, 95% CI: 1.99, 2.17) were twice more likely aware of hypertension compared to those living in Basketo. The odds of awareness of hypertension screening were nearly twice more likely among women living in urban area (AOR: 1.74, 95% CI: (1.69, 1.79) compared to rural.

Women in the age group of 25–34 (AOR: 1.40, 95% CI: 1.36. 1.44) and ≥35 (AOR: 1.19, 95% CI: 1.15, 1.23) years were more aware of hypertension screening than women aged 15–24 and the difference is statistically significant. Women with primary education level were more likely aware for screening of hypertension with (AOR: 1.12, 95% CI: 1.08, 1.15). On the other hand, women with unable to read & write and women who can read and write had no statistically significant association with hypertension screening.

Married (AOR: 1.13, 95% CI: 1.05, 1.22) and single (AOR: 1.28, 95% CI: 1.16, 1.40) women were more likely aware of screening for hypertension than widowed/divorced with significant statistical association. Level of awareness for hypertension screening varies with women's occupational status. Housewives (AOR: 0.64, 95% CI: 0.62, 0.67) and merchants (AOR: 0.58, 95% CI: 0.55, 0.61) were less likely aware of screening for Hypertension compared with other occupations such as students, daily laborers and maidservants. Being government employee were twice more likely aware of hypertension screening with (AOR: 1.99, 95% CI: 1.86, 2.13) compared to others.

Women from households with ≤4 dwellers were 10% less likely aware of hypertension screening compared to women from households with >4 dwellers (AOR: 0. 90, 95% CI: 0. 88, 0. 93). Women from households with middle wealth status (AOR: 1.09, 95% CI: 1.05, 1.13) were more likely aware of screening for hypertension. On the other hand, women from the poor (AOR: 0.96, 95% CI: 0.93, 0.99) and rich family (AOR: 0.86, 95% CI: 0.83, 0.89) were less likely aware of screening for hypertension compared to the richest. However, women from the poorest family had no significant association (Table 2).

### Factors associated with awareness of diabetes screening

Overall, 42.4% of the study participants were aware of screening for diabetes with 44.0% from Gofa and 20.0% from Basketo zone. Women from Gofa zone were three times more likely aware of diabetes screening compared to women in Basketo with (AOR: 3.26, 95% CI: 3.09, 3.44). Women in urban residence were twice more likely aware of screening for diabetes with (AOR: 2.11, 95% CI: 2.05, 2.18).

Women in the age groups of 25–34 (AOR: 1.31, 95% CI: 1.27, 1.34) and ≥35 years (AOR: 1.23, 95% CI: 1.19, 1.28) were more likely aware of diabetes screening. Women who were having primary (AOR: 1.18, 95% CI: 1.14, 1.21) and secondary education (AOR: 1.20, 95% CI: 1.16, 1.24) were more likely aware of screening for diabetes. On the other hand, women who can read and write had no statistical association with awareness of diabetes screening.

Married (AOR: 1.54, 95% CI: 1.42, 1.67) and single women (AOR: 1.53, 95% CI: 1.39, 1.69) were more likely aware of screening for diabetes than widowed/divorced. Being government employee were about two times more likely aware of diabetes screening with (AOR: 1.70, 95% CI: 1.59, 1.82). However, housewives and merchant were less likely aware of screening for diabetes with (AOR: 0.66, 95% CI: 0.64, 0.69) and (AOR: 0.53, 95% CI: 0.51, 0.56) respectively.

Awareness of diabetes screening for women's having family size ≤4 dwellers were 16% less likely aware of screening for diabetes compared to women having with >4 dwellers with (AOR: 0.84, 95% CI: 0.81, 0.86). Women from the poorest (AOR: 0.90, 95% CI: 0.87, 0.94), the poor (AOR: 0.87, 95% CI: 0.84, 0.90), middle (AOR: 0.87, 95% CI: 0.84, 0.91) and the rich households (AOR: 0.76, 95% CI: 0.74, 0.79) were less likely aware of screening for diabetes compared to richest. Women who did not use mass media were more likely aware of screening for diabetes (AOR: 1.07, 95% CI: 1.04, 1.10) compared to those who use mass media (Table 3).

### Factors associated with awareness of cervical cancer screening

In the present study, 38.1% of the study participants were aware of screening for cervical cancer with 40.1% from Gofa and 10.1% from Basketo zone. Women from Gofa zone were about six times more likely aware of cervical cancer screening compared to women from Basketo (AOR: 6.15, 95% CI: 5.74, 6.59). About 58% of urban residents and 34.3% of rural women were aware of screening for cervical cancer. Women in urban residence were twice more likely aware of screening for cervical cancer (AOR: 2.11, 95% CI: 2.03, 2.16).

Women in the age group of 25–34(AOR: 1.51, 95% CI: 1.45, 1.54) and ≥35 (AOR: 1.32, 95% CI: 1.27, 1.36) years were more likely aware of screening for cervical cancer compared to women in the age group15–24 years. Women who can read and write (AOR: 1.26, 95% CI: 1.21, 1.31), have primary (AOR: 1.48, 95% CI: 1.44, 1.53) and secondary school education (AOR: 1.46, 95% CI: 1.41, 1.52) were more likely aware of screening for cervical cancer compared with illiterate women.

Married (AOR: 1.15, 95% CI: 1.06, 1.24) and single women (AOR: 1.64, 95% CI: 1.48, 1.81) were more likely aware of screening for cervical cancer than widowed/divorced. Being government employee were more likely aware for cervical screening (AOR: 1.58, 95% CI: 1.48, 1.69) compared to other occupations. However, housewives (AOR: 0.82, 95% CI: 0.79, 0.86) and merchants (AOR: 0.66, 95% CI: 0.63, 0.69) were 18% and 34% less likely aware of cervical cancer screening respectively.

Women from households having ≤4 dwellers were 18% less likely aware of screening for cervical cancer compared to those with >4 dwellers (AOR: 0.82, 95% CI: 0.81, 0.84). Women from households with the poorest (AOR: 0.84, 95% CI: 0.81, 0.88), the poor (AOR: 0.83, 95% CI: 0.81, 0.86), middle (AOR: 0.90, 95% CI: 0.87, 0.93) and the rich wealth status (AOR: 0.67, 95% CI: 0.65, 0.69) were 16%, 17%, 10%, and 33% less likely aware of screening for cervical cancer compared to the richest respectively (Table 4).

### Factors associated with awareness of breast cancer screening

In the present study, 34.8% of the study participants were aware of screening for breast cancer with 36.6% from Gofa and 10.0% from Basketo zone. Women from Gofa were more likely aware of breast cancer screening compared to women in Basketo zone with (AOR: 5.64, 95% CI: 5.26–6.05). Urban residents were 2.4 times more likely aware of screening for breast cancer compared to the rural (AOR: 2.29, 95% CI: 2.23, 2.36).

Women in the age group of 25–34 (AOR: 1.47, 95% CI: 1.43, 1.52) and ≧35 years (AOR: 1.26, 95% CI: 1.21, 1.30) were more likely aware of screening for breast cancer compared to those aged 15–24 years. Women who can read and write (AOR: 1.08, 95% CI: 1.04, 1.13), had primary (AOR: 1.32, 95% CI: 1.28, 1.36) and secondary education (AOR: 1.22, 95% CI: 1.17, 1.26) were more likely aware of screening for breast cancer compared to illiterate women.

Married women (AOR: 1.40, 95% CI: 1.29, 1.52) and single women (2.17, 95% CI: 1.96, 2.40) were more likely aware of screening for breast cancer compared to widowed/divorced. Government employee were nearly twice more likely aware of screening for breast cancer compared to other occupations such as maidservants, daily laborers and students (AOR: 1.75, 95% CI: 1.64, 1.87). However, housewives (AOR: 0.75, 95% CI: 0.72, 0.78) and merchants (AOR: 0.56, 95% CI: 0.53, 0.59) were less likely aware of screening for breast cancer.

Women from households having ≤4 dwellers were 17% less likely aware of screening for breast cancer compared to those having >4 dwellers (AOR: 0.83, 95% CI: 0.81, 0.85). Women from rich households were 17% less likely aware of screening for breast cancer compared to the richest (AOR: 0.82, 95% CI: 0.79, 0.86). However, women from households with the poorest, poor and middle wealth status were not statistically associated.

Women from distance having less than one hour (AOR: 1.23, 95% CI: 1.17, 1.29) and one to two hours (AOR: 1.11, 95% CI: 1.06, 1.17) were more likely aware of breast cancer screening compared to those travelling greater than two hours to reach the nearest health facility (Table 5).

### Factors associated with awareness of NCD screening

About fifty five percent of the study participants were aware of non-communicable disease screening. Urban women were more aware of screening for NCD (68.2%) than the rural (52.2%) (Table 6). Urban women were statistically associated with awareness of screening for NCD compared to the rural (AOR: 1.68, 95% CI: 1.63, 1.73). Women living in Gofa zone were twice more likely aware of NCD screening compared to Basketo zone (AOR: 2.04, 95% CI: 1.95, 2.13). Women in the age group of 25–34 (AOR: 1.45, 95% CI: 1.41, 1.49) and ≥35 years (AOR: 1.22, 95% CI: 1.18, 1.26) were more likely aware of NCD screening compared to women in the age group of 15–24 years.

Women who can read and write (AOR: 1.06, 95% CI: 1.02, 1.11), have primary (AOR: 1.13, 95% CI: 1.09, 1.16) and secondary education (AOR: 1.11, 95% CI: 1.00, 1.14) were more likely aware of screening for NCD compared to illiterate women. Married (AOR: 1.25, 95% CI: 1.16, 1.35) and single women (AOR: 1.31, 95% CI: 1.18, 1.43) were more likely aware of screening for NCD compared to widowed/divorced.

Housewives (AOR: 0.62, 95% CI: 0.59, 0.64) and merchants (AOR: 0.61, 95% CI: 0.58, 0.64) were less likely aware of screening for NCD than other occupations. Government employees were 63% more likely aware for NCD screening (AOR: 1.65, 95% CI: 1.54, 1.77). Women who had family size greater than four were 8% less likely aware of screening for NCD (AOR: 0.92, 95% CI: 0.91, 0.95) than women who had family size less than four. Women who had no family member living with NCD were 15% less likely aware of screening for NCD compared to women who had family member living with (AOR: 0.86, 95% CI: 0.82, 0.91). Women from households with the poorest (AOR: 0.95, 95% CI: 0.92, 0.99), the poor (AOR: 0.91, 95% CI: 0.88, 0.94) and the rich wealth status (AOR: 0.82, 95% CI: 0.79, 0.85) were less likely aware of screening for NCD compared to the richest. However, women from households with the middle wealth status had no significant association (Table 7).

**Table 7:** Association between awareness of NCD screening and statistically significant variables in the final multiple logistic regression model among 15–49 years women.

Variables	Awareness of NCD screening	*P*-value	*N* = 1,404 AOR (95%CI)	*P*-value
	*N* = 1,404 COR (95%CI)			
Zone				
Gofa	**1.91** (**1.82, 1.98)**	**<0**.**001**	**2.04** (**1.95, 2.13)**	**<0**.**001**
Basketo	1		1	
Residence				
Urban	**1.97** (**1.9, 2.03)**	**0**.**001**	**1.68** (**1.63, 1.73)**	**<0**.**001**
Rural	1		1	
Age				
15–24	1		1	
25–34	**1.37** (**1.33, 1.40)**	**<0**.**001**	**1.45** (**1.41, 1.49)**	**<0**.**001**
≥35	**1.14** (**1.11, 1.18)**	**<0**.**001**	**1.22** (**1.18** **<** **1.26)**	**<0**.**001**
Education level				
Illiterate	1		1	
Read and write	**1.11** (**1.06, 1.14)**	**0**.**001**	**1.06** (**1.02, 1.11)**	**0**.**006**
Primary education	**1.04** (**1.01, 1.07)**	**0**.**003**	**1.13** (**1.09, 1.16)**	**<0**.**001**
Secondary education	**1.22** (**1.19, 1.26)**	**0**.**001**	**1.11** (**1.0, 1.14)**	**<0**.**001**
Marital status				
Married	0.95 (0.88, 1.03)	0.196	**1.25** (**1.16, 1.35)**	**<0**.**001**
Single	**1.25** (**1.20, 1.40)**	**0**.**001**	**1.31** (**1.18, 1.43)**	**<0**.**001**
Widowed/divorced	1		1	
Occupation				
Housewife	**0.62** (**0.60, 0.64)**	**0**.**001**	**0.62** (**0.59, 0.64)**	**<0**.**001**
Merchant	**0.61** (**0.60, 0.64)**	**0**.**001**	**0.61** (**0.58, 0.64)**	**<0**.**001**
Gov't Employee	**1.50** (**1.40, 1.60)**	**0**.**001**	**1.65** (**1.54, 1.77)**	**<0**.**001**
Other	1		1	
Family size				
≤4 dwellers	**0.92** (**0.9, 0.94)**	**0**.**001**	**0.92** (**0.91, 0.95)**	**<0**.**001**
>4dwellers	1		1	
Family member living with NCD				
No	**0.72** (**0.70, 0.75)**	**0**.**001**	**0.86** (**0.82, 0.91)**	**<0**.**001**
Yes	1		1	
Wealth status				
Poorest	**0.81** (**0.79, 0.84)**	**0**.**001**	**0.95** (**0.92, 0.99)**	**0**.**007**
Poor	**0.78** (**0.76, 0.81)**	**0**.**001**	**0.90** (**0.87, 0.93)**	**<0**.**001**
Middle	**0.94** (**0.91, 0.97)**	**0**.**001**	1.00 (0.97, 1.04)	0.760
Rich	**0.79** (**0.77, 0.82)**	**0**.**001**	**0.82** (**0.79, 0.85)**	**<0**.**001**
Richest	1		1	

COR, crude odds ratio: odds ratio by bivariate analysis, 95% CI, confidence interval at the 95% level.

AOR, adjusted OR: odds ratio by multiple logistic regression 1: referent category.

Bold values indicate statistical significance at *p*-value ≤0.05.

## Discussion

Awareness of screening and early diagnosis of diseases significantly affecting utilization of services (18, 21). However, less attention has been paid in the post-pandemic era of COVID-19. Therefore, this study aimed to assess awareness of NCD screening and associated factors among reproductive-age women. This information could be useful in designing strategies to improve utilization of screening services which leads to early diagnosis and treatment, and prevents risk of complications and future mortality.

In this study, the level of awareness for screening NCDs among reproductive age women was found to be 54.8%. Specifically, the percentages for awareness of hypertension (HTN), diabetes, cervical cancer, and breast cancer screenings were 52.7%, 42.4%, 38.1%, and 34.8% respectively. However, the study revealed that only 43.0%, 9.4%, 16.2%, and 20.7% of the participants had undergone screening for HTN, diabetes, breast cancer, and cervical cancer respectively.

More than half of the study population had awareness of screening for hypertension. The study conducted in rural Puducherry of India showed that 73.3% of women were aware of screening for hypertension (27). This might be due to the difference in the setups. Utilization of healthcare services and frequency of contact with health workers might be higher in India due to relative advantage of the setting. The frequent contact with health workers might favor the high prevalence of awareness of hypertension screening among women.

Women in the age groups of 25–34 and ≥35 years were more likely aware of screening for hypertension screening compared to women aged 15–24 years. This study is in line with study conducted in Puducherry, India (27). In this study, the awareness of screening for hypertension was higher in urban areas compared to rural areas. This might be due to the difference in accessing information related to hypertension screening.

Level of awareness for hypertension screening varies with women's marital status. Being married and single women were more likely associated with screening of hypertension compared to Widowed/divorced/separated. This study is in line with study conducted in rural Puducherry of India that being married was independently associated with awareness of screening for hypertension (27). This is also supported by a study conducted in North Shewa Zone Oromia region, Ethiopia. Being widowed/divorced was found to have inadequate knowledge of non-communicable disease including hypertension. Thus, being widowed was associated with worse health outcomes (28).

Level of education was another background characteristic that had significant influence on awareness about hypertension screening services. Women who can read and write, women who have primary and secondary education were associated with a higher rate of awareness of hypertension screening compared with illiterate, and this ﬁnding is consistent with the results of Russian study that highlighted being low-educated was associated with lower rates of awareness of hypertension (29). Access to healthcare might explain the high rate of awareness among people with education and low educational status is a stronger marker of low hypertension awareness (30). Government employees were more likely aware of screening for hypertension screening. This finding agrees with study conducted in India that employed women were more aware of screening for hypertension (27).

Women from households with the poor and rich wealth status were less likely aware for hypertension screening compared to the richest wealth status. Consistent finding was reported in study conducted in South Asia that awareness of hypertension screening was low in the household with low wealth status (30).

Overall, 42.4% of the study participants were aware of screening for diabetes with 44% from Gofa zone and 20% from Basketo special Woreda. However, this finding is lower than study conducted in rural Puducherry of India. The study showed that 73% of women were aware of screening for diabetes (27). This discrepancy might be explained difference in the setting, sample size as well as study design used. Women in the age groups of 25–34 and ≥35 years were more likely aware of screening for diabetes compared to those aged. Similarly, the result of a study conducted in Syria reported that older age was associated with awareness of diabetic screening (31).

Over sixty percent of urban women were aware of screening for diabetes and they were more likely aware of screening for diabetes. The finding was in line with a study conducted in Saudi Arabia that showed urban residents had higher awareness rates than rural (32). In this study, women who can read and write, have primary and secondary school education were more likely aware of screening for diabetes compared to those with illiterate women. Consistently, a study conducted in Imo and Kaduna states, Nigeria showed that women's level of education had statistically significant association with awareness of diabetes screening (33). Married and single women were more aware of screening for diabetes compared to widowed/divorced women with significant statistical association.

Being government employee was more likely aware for diabetes screening. However, housewives and merchants were less likely aware of screening for diabetes. This finding agree with a study conducted in Syria showed that being employed were significantly associated with increased level of awareness for diabetic screening (31). Women from households with the poorest, poor, middle, and rich wealth status were less likely aware of screening for diabetes compared to richest women. Consistent finding was reported in study conducted in Syria (31).

Awareness of cervical cancer varies across developing countries. In the present study, 38.1% of the study participants were aware of screening for cervical cancer with 40.1% from Gofa and 10.1% from Basketo zone. The finding is higher than studies conducted in India (35%) and Southern Ghana (31.6%) (27, 34). In contrast, it is significantly lower than studies conducted in Nigeria 64.3% (35) and Southern Ghana 68.4% (34). This difference could be explained in the difference in access of information among the study settings. Women in urban residence were more likely aware of screening for cervical cancer compared to rural residence and independently associated with awareness of screening for cervical cancer. The reason could be due to limited information access among women living in the rural area.

Current study shows that awareness of cervical cancer screening is positively associated with women's level of educational. Women, who can read and write, have primary and secondary school education were more likely aware of cervical cancer screening compared to illiterate women. The finding is in line with a study in Dessie town that showed awareness of cervical cancer screening increases with the level of women's educational status (36). Similar finding was reported in a study conducted in Zambia (37). This might be attributed to better interest and access to resources and information that more educated women could have. Researchers have argued that educating women on the importance of cervical cancer screening and on ways of preventing cervical cancer will increase their awareness and also reduce the prevalence of cervical cancer. Suitable and significant steps should be taken to inform women about the advantages of cervical cancer screening tests (19).

Women in the age groups of 25–34 and ≥35 years were more likely aware of cervical cancer screening than women aged 15–24 years. This finding aggresses with a study conducted in Zambia (37) and awareness of cervical cancer screening was also highest among women aged 25–34 years in Benin and women aged 45+ in Cameroon (38). Studies show that as a woman's age increases, she will possibly be more aware of the health issues that are associated with her reproductive health (39).

Married and single women were more likely aware of screening for cervical cancer than widowed/divorced with significant statistical association. Similarly finding was reported in demographic health survey report of Benin and Cameron that, currently/formerly married women were more aware of screening than those who were never in a union in Benin and Cameroon (38). Government employees were more likely aware for cervical screening compared to other occupations. However, women with housewife and merchant were less likely aware of screening for cervical cancer. This finding is in line with a study conducted in Benin and Zimbabwe that having a professional/technical/managerial occupation significantly increased the odds of awareness of cervical cancer (39). Women from households having ≤4 dwellers were less likely aware of screening for cervical cancer compared to those having more than four dwellers. This finding contrasts with study done in Tanzania that having 1–4 children were more likely aware of screening for cervical cancer (40). The difference could be attributed by variation in socio-cultural factors between the two study areas.

Awareness of cervical cancer differed by wealth quintiles in this study. Women from households with the richest (44.2%), the rich (33.7%), the middle (39.7%), the poor (36.9%) and the poorest wealth status (36.4%) were aware of screening of cervical cancer with significant statistical association. Women from the poorest, the poor, middle and the rich households were less likely aware of screening for cervical cancer compared to richest women. Another similar study in Ethiopia reported that women with a higher average monthly income had better awareness of cervical cancer screening (40). The finding from Benin (20.8%) and Cameroon (71.0%) showed that women in the richest wealth quintile had also the highest proportion of awareness (38). This might be related with high-resource settings, education level, and socioeconomic status predicts awareness of cervical cancer screening.

Awareness of breast cancer screening is important for success of prevention intervention. In the present study, 34.8% of the study participants were aware of screening for breast cancer. This finding is lower than study conducted in Addis Ababa, Ethiopia (53.1%) (41) and India 45% (27). The difference could be attributed to the higher educational status in study settings such as Addis Ababa city and India compared to ours. Women in the age groups of 25–34 and ≥35 years were more likely aware of screening for breast cancer compared to women aged 15–24 years. The finding was consistent with a previous study conducted in china (42) that showed increasing age of women was significantly associated with the awareness of breast cancer screening.

Women who were who can read and write, have primary and secondary school education were more likely aware of screening for breast cancer compared with illiterate women. This might be, as women's educational level increases, it may correlates with improved socio-economic status, thus eliminating key barriers to the acquisition of health information and healthcare services.

Our study revealed that married and single women were more likely to be aware of breast cancer than those widowed/divorced separated women. This result was consistent with previous research findings (43) where married women had better breast cancer awareness. The finding could be attributed to the fact that married women might have received encouragement and motivation from their partners to seek health care and therefore might have been exposed to breast cancer education. Government employees were more likely aware of breast cancer screening compared to other occupations. However, housewives and merchants were less likely aware of screening for breast cancer. Consistent with previous research (44) that showed occupation income can influence women's awareness level for breast cancer screening.

Household income is associated with the awareness of breast cancer screening. Women from households with the rich wealth status were less likely aware of screening for breast cancer screening compared to the richest. When a family has better income it will also increase access to education and information. This finding is consistent with community based study in Addis Ababa, Ethiopia (41). Women from distance taking less than one hour and 1–2 h to reach the nearest health facility with were more likely aware of breast cancer screening compared to those from distance taking greater than two hours. Similar finding was reported in study conducted in china that showed living within a short distance from the nearest health facility was associated with breast cancer screening (45).

Over half of the study participants heard/read about non communicable disease screening. The current study is consistent with a study from Bangladesh (57.9%) (46). In contrast, the ﬁnding of this study is higher than the study done in Switzerland (47). The variation might be due to differences in socio-demographic characteristics of the study population and sampling techniques. Our study showed that women from Gofa zone were twice more likely aware of NCD screening compared to Basketo zone residents. The difference could be attributed to the socioeconomic variation between the two zones. When there is a better socioeconomic status, it will also increase access to education and information. In the current study, married and single women were found to be more aware of NCD screening. The finding is consistent with a study conducted in India and America that showed widowed divorced had inadequate knowledge of NCDs. As a result, being widowed was associated with worse health outcomes (28, 48). Women, who can read & write, have primary and secondary school education were more likely aware of screening for NCDs compared to illiterates. This finding agrees with a study conducted in India (27) that reported women below primary level of education were independently associated with unawareness of screening for NCDs.

In a current study, having a family member with NCD(s) had increased the likelihood of NCDs awareness. This is also evidenced by previous studies (49, 50). This might be due to study subjects' involvement in caregiving to family members' with NCDs (50). Moreover, receiving information from health professionals increased the chance of having adequate knowledge of non-communicable diseases (49) Receiving counseling services from health care providers will also give opportunity for discussion on various health topics (49) and result in increased awareness and understanding of non-communicable disease. Women from households with the poorest, the poor and the rich wealth status were less likely aware for non-communicable disease screening compared to the richest. The finding agrees with study conducted in higher Myanmar (50). This might be due to better access that women with higher wealth status could have to information and education related NCD screening.

### Strengths and limitations

This pionering study in Gofa and Basketo zones examines the level of awareness and factors linked to awareness of NCD screening among reproductive-aged women. Its strengths lie in its community-based approach covering both rural and urban residents, ensuring broader applicability. Additionally, rigorous measures were implemented, including pilot testing of instruments, comprehensive training of data collectors and supervisors, and high response rates (99%), and enhancing data quality. The study's use of weighted analysis allows for extrapolation of findings to the entire study area. It delves into socioeconomic and knowledge-related factors associated with risk factors. However, the study's cross-sectional design limits its ability to establish causal relationships.

## Conclusion

The study revealed that, 45.2% of the study population had no awareness of non-communicable disease screening and the vast majorities were left unscreened for the diseases. This low awareness of screening for NCDs including diabetes, HTN, breast cancer and cervix highlights the need to target more towards younger age (15–24), housewife, merchant, women living in households having family size ≤4 dwellers, low economic status, Basketo zone and rural residents. Awareness of screening for NCDs such as diabetes, cervical and breast cancer should also target to women who have lower education. Government need to work on improving access and availability of screening services. Policy makers in the health sector including health development partners need to strengthen health system and design strategies to foster screening program for NCDs. Healthcare providers need to work on improving awareness of NCDs screening through mass media campaigns and health education programs among reproductive age women.

## Data Availability

Most of the data used to support the findings of this study are included within the article. More data is available upon reasonable request. The data can be provided through corresponding author whenever reasonably requested.
